# Improving the Network Scale-Up Estimator: Incorporating Means of Sums, Recursive Back Estimation, and Sampling Weights

**DOI:** 10.1371/journal.pone.0143406

**Published:** 2015-12-02

**Authors:** Patrick Habecker, Kirk Dombrowski, Bilal Khan

**Affiliations:** Department of Sociology, University of Nebraska-Lincoln, Lincoln, Nebraska, United States of America; University of Westminster, UNITED KINGDOM

## Abstract

Researchers interested in studying populations that are difficult to reach through traditional survey methods can now draw on a range of methods to access these populations. Yet many of these methods are more expensive and difficult to implement than studies using conventional sampling frames and trusted sampling methods. The network scale-up method (NSUM) provides a middle ground for researchers who wish to estimate the size of a hidden population, but lack the resources to conduct a more specialized hidden population study. Through this method it is possible to generate population estimates for a wide variety of groups that are perhaps unwilling to self-identify as such (for example, users of illegal drugs or other stigmatized populations) via traditional survey tools such as telephone or mail surveys—by asking a representative sample to estimate the number of people they know who are members of such a “hidden” subpopulation. The original estimator is formulated to minimize the weight a single scaling variable can exert upon the estimates. We argue that this introduces hidden and difficult to predict biases, and instead propose a series of methodological advances on the traditional scale-up estimation procedure, including a new estimator. Additionally, we formalize the incorporation of sample weights into the network scale-up estimation process, and propose a recursive process of back estimation “trimming” to identify and remove poorly performing predictors from the estimation process. To demonstrate these suggestions we use data from a network scale-up mail survey conducted in Nebraska during 2014. We find that using the new estimator and recursive trimming process provides more accurate estimates, especially when used in conjunction with sampling weights.

## Introduction

Due to the difficulty of studying hidden and hard-to reach populations, many researchers have moved past large general surveys to develop a specialized set of data collection methods. These techniques include observation, participation, key informant interviews, and location-based strategies that can provide valuable insight about population characteristics, but which rarely generate measures of representativeness of the sample or the size of the population as a whole. The latter is important for a range of concerns, from policy to research methodologies. The development of methods such as respondent-driven sampling [1–3] and venue-based sampling [4,5] gives researchers ways to gather semi-representative data about many hidden populations, but recent work in this area has shown the importance of knowing overall population size for the estimation of sample characteristics [6,7]. Although an improvement over convenience sampling, techniques for reaching hidden populations remain considerably more expensive and time-consuming than many general population sampling strategies.

The network scale-up method (NSUM) is a technique designed to generate size estimates for hard-to-reach and hidden populations without having to directly interview or send a survey to a member of the target group. Rather, the estimation process takes advantage of conventional sampling frames to recruit a representative sample of the larger population of which the hidden population is a part. This sample is then asked to estimate the number of people each respondent knows who would fall into the hidden population in question. This approach has two major advantages. First, NSUM methods do not ask respondents about their own characteristics. Stigmatized or hidden populations may be reluctant to disclose their own status due to perceived risk, even in an anonymous survey. Rather, by limiting questions to the anonymous enumeration of contacts, the hope is that second-hand reporting will lessen the burden of stigma on those involved in providing project data. Second, because we do not need to directly interview members of a hidden population, we can return to general sampling techniques such as random digit dialing or addressed based sampling, survey methods that are considerably cheaper and easier to implement and which take advantage of established sampling frames.

In recent years, NSUM techniques have been used to estimate the prevalence of HIV/AIDs [8,9], deaths after an earthquake [10], the size of MSM (i.e. men who have sex with men) populations [11,12], and other hard-to-enumerate populations [13,14]. The increasing number of NSUM projects and the variety of populations/locations where it has been implemented demonstrates the flexibility of the method. Questions remain, however, about both the potential and the limits of the technique. As the method becomes more popular, developing new versions of the estimator and ways to improve the accuracy of their estimates will become increasingly important.

We propose a step in this direction via a new form of the NSUM estimator and a novel, recursive trimming process that is applied to the set of study variables that are used as the basis of the scale-up estimates. Additionally, we demonstrate how to incorporate sampling weights into the NSUM estimation process. Using data collected in 2014 from an address-based random sample of Nebraska, we show how estimates evolve via the iterative trimming of poor performing elements of the estimator. By improving the overall accuracy of the remaining set of NSUM predictors, this recursive process provides more accurate and consistent estimation within the NSUM framework.

## The Original Network Scale-Up Estimator

The network scale-up estimation method is based on the assumption that, on average, an individual’s personal network will be representative of the general population [8,15]. That is, the proportion of people in an average individual’s personal network who are members of a given subpopulation is indicative of the relative size of that subpopulation to the general population as a whole. This can be formally expressed with Eq 1, where *m* is the number of people known by the respondent in a given subpopulation, *c* is the size of the respondent’s personal network, *t* is the size of the larger population, and *e* is the size of a subgroup in the population.

$$\frac{m}{c}=\frac{e}{t}$$ (1)

The challenge of the NSUM method is estimating the size of an individual’s personal network, *c*. Realizing that local conditions can influence mean network size (consider the difference between predominantly urban and predominantly rural populations) a popular method for calculating this value for a sample is the *known population method* [14]. This approach asks respondents to report the number of individuals they know from a population whose size *can* be estimated by other means (e.g. Census figures or other official statistics). These data can then be used to estimate the personal network size of each respondent, allowing researchers to “scale up” their answers for unknown populations to population level estimates. Eq 2 describes how the counts for such “scaling” variables can be used to derive the personal network size of a single respondent, where *i* indicates a respondent and *j* a scaling variable. In essence, the reported value of each scaling variable *m*_*ij*_ (say, “firefighters”, or “airline pilots,” or “persons named Walter”) are summed across a range of such categories, and then divided by the total known population *e*_*j*_ for these same groups.

$${\hat{c}}_{i}=\frac{\sum_{j}m_{ij}}{\sum_{j}e_{j}}t$$ (2)

Common populations to ask about include the number of people with a given first name, such as Rose, or the number of people known who hold a certain job, such as postal worker; “knowing someone” is normally defined as someone whom the respondent knows by name and with whom the respondent has had some form of communication in the past two years [13].

Once an estimate for the respondent’s personal network size is in hand it is possible to calculate the size of a previously *unknown* subpopulation using the ratio of the respondents estimated personal network size to the total population. This is shown in Eq 3 (where data solicited from all respondents (0,1,K *i*) for a given “target” population *j*, over the sum of all respondent’s respective, estimated personal network size ${\hat{c}}_{i}$, is used to estimate the number of people in the target population (such as illegal drug users).

$${\hat{e}}_{j}=\frac{\sum_{i}m_{ij}}{\sum_{i}{\hat{c}}_{i}}t$$ (3)

The standard error of Eq 3 can be expressed as:

$$s.e.\left(\hat{e}\right)=\sqrt{\frac{{\hat{e}}_{j}}{\sum_{i}{\hat{c}}_{i}}t}$$ (4)

The NSUM method carries three main assumptions beyond our initial assumption that an average individual’s personal network will be proportionally representative of the general population [14].

- *There are no barrier effects*, i.e. that everyone in the larger population has an equal probability of knowing someone in a given subpopulation. A national survey interested in the number of people who are attacked by sharks may suffer barrier effects as respondents who live in Montana may have different probabilities of knowing a shark attack victim than a respondent living in Florida.
- *There are no transmission effects*, i.e. everyone is fully aware of the characteristics that define a given subpopulation. For instance, if a researcher is interested in estimating the number of people who have been in prison in the last 30 days, the method assumes that the survey respondent is fully aware of any persons in their personal network who have been to prison in the last 30 days. If someone in their personal network did go to prison, but the respondent is unaware, the result would be an underestimate of the size of that population if this were a target variable, or an underestimation of personal network size if this was a scaling variable. Violations of this assumption are more likely when the target group is associated with a stigma or when the attribute of the group is something that is rarely discussed even with close friends and family.
- *Respondents can correctly recall the number of people that they truly know in the subpopulation*, and can do so in the timeframe allowed by the study. Different types of survey modes could be expected to result in different recall effects. An interview on the phone may not provide the respondent enough time to adequately complete a full response process, where a mail survey allows respondents time to think and check other sources of information, although this latter option is not always desirable. The expected exposure of the target population may also alter recall effects. If the population in question is sufficiently common, it may be difficult for the respondent to accurately enumerate the number of people they truly know and they may simply guess at an appropriate range (e.g. answers to how many people do you know who eat fast food at least once a month).

Provided these assumptions can be justified, the estimation process for gauging the size of a hidden population can be reduced to a series of questions about the number of persons known to be members of a range of scaling populations and the number of persons they know in the target population(s). When asked of a sample of respondents drawn from a conventional sampling frame, researchers can harness the advantages that come from working in known sampling scenarios, such as conventions around the treatment of outliers, and the weighting of outcomes according to sampling results (both of which were carried out here).

With this in mind, we note that in the method above, the final value for the scaling variables determined in each survey are not treated individually. Rather, as originally practiced both the discovered variable values across all respondents, *m*_*ij*_, and the total (external) estimates of these “known” populations, *e*_*j*_, are summed (see Eq 2). The resulting ratio is used to calculate an individual’s personal network size. In this process, large estimates in one scaling variable *m*_*i*_ are minimized in their ability to alter the resulting personal network size estimate, given relative uniformity across the other scaling variables. Further, by summing across the known network sizes, *e*_*j*_, differences in the sizes of these known populations introduce a hidden weighting factor, whereby some variables contribute more to the size of the denominator than others. The latter problem is often dealt with by seeking scaling variables that are roughly equal in estimated size i.e. where *e*_*a*_ ≈ *e*_*b*_ ≈ *e*_*c*_ ≈…≈ *e*_*j*_, in order to minimize the hidden weighting that unequal sizes entails. Further, in a situation where no means are available to discover outliers and remove them from the estimation process, we may prefer a method that implicitly mutes the impact of outliers in our estimation process.

Finding scaling variables of uniform size may be difficult, however, and muting the effect of outliers is not the same as removing them from the estimation process. In both cases, alternatives are available. Toward this end, we discuss an alternative estimator that takes into account the performance of the each scaling variable individually and, allows for the selective removal of those that are performing poorly in comparison with the combination of all others. The new estimator and a comparison of results with the original estimation process are discussed below.

We also propose a way to integrate sampling and post-stratification weights into both of the estimation processes. One of the strengths of the NSUM technique is that it can use mainstream sampling techniques to generate representative samples and thereby accurate estimates of target populations. These types of frames also have the major advantage of having known distributions which can be used to create weights to ensure greater representativeness of the sample. Incorporating these types of weights in to the NSUM estimation process is a logical and much needed addition to the technique. Below we demonstrate how weights can be included into both the original and proposed estimators and compare the differences in the final population estimates.

## Current Study

In the spring of 2014 the *Nebraska Community Survey* was sent to a random sample of 2,000 households in Nebraska. The sample was obtained from the United States Postal Service delivery sequence file (DSF) through the Bureau of Sociological Research at the University of Nebraska-Lincoln. Seasonal and vacant households were removed from the sample by the provider. The DSF covers approximately 97 percent of U.S. Households and provides a reasonable frame for the Nebraska population [16,17]. The Institutional Review Board at the University of Nebraska-Lincoln approved the research protocol and granted the project an exempt status (IRB# 20140314288 EX). Each household in the sample was sent a packet which included a letter introducing the survey, a one dollar incentive, a copy of the survey questionnaire, and a pre-paid return envelope. The person in the household to take the survey was selected using the next-birthday method, a quasi-probability selection design [18]. Eligible respondents had to be at least 19 years old, the age of majority in Nebraska, and be the next person in the household to have a birthday after April 14, 2014. A week after the initial mailing a reminder postcard was sent to any households that had not responded to the initial mailing. Three weeks later, a second survey packet was sent to non-responding households. If at any time a respondent asked to be removed from the address list all further mailings were stopped. After the third mailing data was collected for approximately another six weeks, allowing respondents ample time to complete and return their survey.

The goal of this survey was to gather data about a wide variety of hidden and difficult to measure populations. Several researchers at the University of Nebraska-Lincoln came together and developed a list of outcomes that ranged from domestic migration in the US and Nebraska, public health concerns, drug use, contact with the criminal justice system and crime victimization. The NSUM technique allows for the easy incorporation of research questions that can be measured in counts of persons known to the respondent. Due to the unusual nature of the NSUM questions survey materials were written to emphasize the unique aspects of the survey. At the point of data collection cutoff approximately 31 percent of the surveys had been completed and returned, providing an analytic sample of 618. Our NSUM survey achieved a higher response rate than similarly incentivized mail surveys of Nebraskans in the same field timeframe, an increase we attribute to the novelty of the NSUM approach.

Item non-response, or individual questions that have missing data in an otherwise complete survey, is somewhat challenging with pen and paper NSUM surveys. Several respondents appeared to favor only writing answers when they had non-zero responses to enumeration questions, leaving large and seeming random numbers of questions blank, even though they completed the survey, and supplied no “0” answers for any of the questions. Faced with this situation, we had two general choices: a conservative approach where we considered blank answers to be missing and thus handled through standard practice such as listwise deletion or multiple imputation; or assume that the empty cell indicates that the respondent knows zero people for that question and substitute a zero for the missing value code. As this latter option infers respondent behavior that cannot be confirmed, we view this as a weaker assumption and did not implement this treatment of item non-response for count measures. As a result, the final sample of “mostly” complete surveys was 550.

This study used the known population approach discussed above to calculate a respondent’s personal network size. Each respondent was asked for counts of personal network members of eighteen populations of known size, including twelve categories of people with a given first name and six professions.

A range of target variables were collected as well. As above, these are populations for which we assume that we have no available, external and reliable source for the size of the population. These populations are estimated using the scaling variables. For purposes of demonstration, in this paper we selected three target variables for estimation: the number of people who had moved to Nebraska from another state in the U.S. in the last two years; the number of people in Nebraska who would not approve of interracial dating; and the number of people in Nebraska who had used heroin in the last 30 days. Each survey respondent was asked to count the number of people they knew in each of these categories, not including themselves, and these counts were used in Eq 3 to create population estimates of the size of these subgroups in the state-wide population. The results using the original NSUM method were that 12,184 people moved to Nebraska from another state in the U.S. in the last two years; there are approximately 17,891 people in the Nebraska who do not approve of interracial dating; and there are an estimated 367 people in Nebraska who have used heroin in the last 30 days.

## The Mean of Sums Network Scale-up Estimator

The heart of the network scale-up estimation process is based around the number of people a respondent knows from a known subpopulation. When there is only one known subpopulation the ratio is simply that, the number of people an individual knows (say, for example, “persons named Walter”), divided by the total size of that subpopulation (in this case, the number of persons named Walter in the population). Network scale-up researchers, however, often use more than one scaling variable (and thus more than one known subpopulation) in building an estimate of personal network size. Recent recommendations include the use of at least twenty [13]. As above, the number of people known across all known subpopulations are summed and taken over the sum of the size of all the subpopulations (as shown in Eq 2).

This method can lead to hidden masking of the performance of a single variable. This may be desirable when there are limited means to judge the performance of the scaling variables individually, but in general the effects on the resulting estimates are not discussed. An example can help make this process more clear. Consider an individual who provides counts for three scaling variables of known population sizes 1000, 1000, and 1000 respectively, who indicates that she knows 1 person in each of these categories. The result (see Eq 5), using the conventional NSUM estimation procedure is that the respondent’s personal network size is

$$\frac{1+1+1}{1000+1000+1000}=0.001$$ (5)

or, 0.1% of the total population. However, if the size of one of the scaling variable’s actual populations is much smaller (say, 100 instead of 1000), and one is much larger (say 10,000 rather than 1000), then the size of this same respondent’s personal network is equal to 0.00027 or 0.027% of the total population (see Eq 6).

$$\frac{1+1+1}{100+1000+10000}=0.00027$$ (6)

Though the number of individuals known remains the same, the differential contribution of the elements of the denominator means that the larger target population (10,000), virtually eliminates the fact that here a person from a rare population is known (1/100). Indeed, it would make little difference if she had reported knowing 2 persons in the first population (2/100). Where significant differences exist in the size of the scaling populations (i.e. the denominator), the significance of knowing individuals in smaller populations can make little difference in the estimate of personal network size. Given this, it might make more sense to take the average of the individual scaling variable ratios (Eq 7, here after the mean of sums (MoS) estimator).

$${\hat{c}}_{i}=\frac{\sum_{j}\frac{m_{ij}}{e_{j}}}{j}t$$ (7)

Here, each scaling variable contributes equally to the final estimate. However, here we find the issue nearly reversed: the smaller population now dominates the sum, and we get a result that is nearly 3 more than 10 times the size estimated using the conventional method (see Eq 8).

$$\left(\frac{1}{100}+\frac{1}{1000}+\frac{1}{10000}\right)/\;3=0.0037$$ (8)

Which of these is a better ordinary method for estimating personal network size remains an open question. Below we show that the MoS estimator performs far better in the recursive trimming process, especially when weights and removal of outliers are incorporated as well.

Table 1 shows the differences in population estimates of our target populations using the original estimator (Eq 3 above) and one derived from an estimator that incorporates the MoS method (Eq 9) for three populations in Nebraska.

**Table 1:** Change in Three Population Estimates and Personal Network Size over the Original and MoS Estimator.

		Original		MoS	
		Estimate (s.e)	95% CI	Estimate (s.e)	95% CI
Moved to Nebraska from within the US in last 2 years		12184	[11673, 12695]	75800	[74821, 76777]
		(260.60)		(499.14)	
Would not approve of interracial dating		17892	[17273, 18510]	22614	[22079, 23148]
		(315.79)		(272.63)	
Heroin use in last 30 days		368	[279, 457]	454	[379, 530]
		(45.28)		(38.64)	
Personal Network Size					
	Mean	604.03		1024.28	
	Standard Deviation	694.04		1559.17	
	Min	0.00		0.00	
	Max	5944.31		16794.19	
	N	555.00		555.00	

$${\hat{e}}_{j}=\frac{\sum_{i}\frac{m_{ij}}{{\hat{c}}_{i}}}{i}t$$ (9)

In this case, the scaling variables were selected to be as close as possible in size given the list of available “known” populations. Toward this end, we used first names which are thought to comprise a similar percentage of the US population [19]. However, we were more constrained in the use of professions, as the list of common, known subpopulation easily identifiable by a mail survey are limited. These ranged from a low of 310 (airline or commercial pilots in Nebraska) to 4943 (police officers in Nebraska).

Differences between these two estimators vary depending upon the target population being estimated. The estimate of the number of people who moved to Nebraska from another state in the U.S. in the previous 2 years increased by a factor of 6 to 75,800 with the MoS estimator. The American Community Survey reports that in the year 2013, 45,854 Nebraskans reported living in a different state 1 year ago [20]. When added to the same report for 2012 of 43,266 people moving into Nebraska [21], this provides a 2 year total of 89,120. The MoS estimate of 75,800 is considerably closer to the 2 year ACS total than the estimate of 12,184 provided by the original NSUM formula, although the 95% confidence intervals for neither estimate contain the ACS statistic. The Internal Revenue Service also compiles state-to-state migration data, but they have not yet released data for 2012 or 2013 at this time. Once that data is available it will provide an additional benchmark to test our estimates against. In addition to an increase in estimate migration into Nebraska, the MoS method also estimates that 22,614 Nebraskans do not approve of interracial dating compared to an estimate of 17,892 Nebraskans from the standard formula. The estimate for the number of Nebraskans that have used heroin in the last 30 days increases from 368 to 454. Not all of the differences in our survey were stark. Overall, of the 46 populations we estimated in the larger study, 76% of them changed by less than a factor of 2 using the MoS estimator compared to the original estimator.

A key indicator of how the results change is shown by the differences in calculated personal network size between the two estimation methods. The average size of the calculated personal network size increases considerably, as does the standard deviation, when using MoS. Perhaps the most dramatic change is the maximum personal network size calculated by both formulas. The largest network size under the MoS estimator is 16,794 while the original estimator peaks at 5,944.

## Using Back Estimates as a Data Quality Check

As the network scale-up method is mainly used to estimate the size of hidden and hard-to-reach populations, it is difficult to gauge the quality of the estimation process. Using back estimation of the original scaling variables can provide some sense of the relative accuracy of the individual estimation variables [8,12,22]. As Guo and colleagues [12] have recently shown, this process can be used as a self-check for the performance of individual scaling variables. The process proposed by these authors is to treat the scaling variables as target variables after the estimates of personal network size have been made. In our survey, this would include the 12 names (i.e. Bruce and Martha) and 6 professions (i.e. firefighters and airline pilots) which are listed in Table 2. In the back estimation process, Eq 3 is used to estimate one of these known categories (say, for example, the number of firefighters in Nebraska), treating it as a target variable. The resulting back estimate can then be compared with the known population of this category. Significant variation between back estimates of target variables and the known values for those populations points to elements in the network size estimation process where respondents deviated most significantly from the expected values, based on the results of all of the other knowns. This process can be used for all versions of the NSUM estimator discussed in this paper, original and MoS, weighted and unweighted.

**Table 2:** Recursive Back Estimation Process to Identify and Eliminate Poor Predictors Using the Original Estimator Without Weights.

		(1)	(2)	(3)	(4)	(5)	(6)	(7)	(8)	(9)	(10)	(11)	(12)	(13)	(14)	(15)	(16)	(17)
Names		Known	IT0	ABS^1^	IT1	ABS^1^	IT2	ABS^1^	IT3	ABS^1^	IT4	ABS^1^	IT5	ABS^1^	IT6	ABS^1^	IT7	ABS^1^
	Walter	7455	1850	2.01	2351	1.67	2499	1.58	2504	1.57	2303							
	Bruce	4914	3929	0.32	4992	0.02	5307	0.11	5317	0.11	4890	0.01	4422	0.15	4500	0.13	4969	0.02
	Alan	3812	4704	0.30	5977	0.65	6354	0.74	6365	0.74	5854	0.62	5293	0.47	5387	0.50	5949	0.64
	Ralph	5269	2196	1.26	2790	0.92	2966	0.83	2972	0.83	2733	0.95	2471	1.09	2515	1.07	2777	0.92
	Kyle	2990	3946	0.40	5014	0.75	5330	0.83	5340	0.84	4911	0.72	4441	0.57	4519	0.60	4990	0.74
	Adam	4839	4754	0.03	6040	0.32	6421	0.41	6433	0.41	5916	0.29	5350	0.14	5445	0.17	6012	0.31
	Rose	5531	2982	0.89	3788	0.55	4027	0.46	4035	0.45	3711	0.58	3355	0.72	3415	0.70	3771	0.55
	Tina	4111	2954	0.48	3753	0.13	3990	0.04	3997	0.04	3676	0.16	3324	0.31	3383	0.28	3736	0.14
	Emily	3887	4587	0.24	5828	0.58	6195	0.67	6207	0.68	5708	0.55	5162	0.41	5253	0.43	5801	0.58
	Martha	7698	2029	1.92	2578	1.58	2740	1.49	2745	1.49	2525	1.61	2283					
	Paula	4055	2553	0.67	3243	0.32	3448	0.23	3454	0.23	3177	0.35	2873	0.50	2923	0.47	3228	0.33
	Rachel	4522	3896	0.21	4950	0.13	5262	0.22	5272	0.22	4848	0.10	4384	0.04	4462	0.02	4927	0.12
Jobs																		
	Police Officers	4943	9542	0.95	12123	1.29	12888	1.38	12912	1.39	11875	1.26	10737	1.12	10928	1.14		
	Firefighters	1200	18248	3.93	23184													
	US Postal Carriers	2170	5808	1.42	7379	1.77	7844											
	Correctional Officers	2490	764	1.71	970	1.36	1031	1.27	1033	1.27	950	1.39	859	1.53	874			
	Licensed Gun Dealers	1159	1466	0.34	1862	0.68	1980	0.77	1984	0.78	1824	0.65	1650	0.51	1679	0.53	1854	0.68
	Airline or Commercial Piolots	310	758	1.29	963	1.64	1024	1.72	1026									

^1^: This value is the absolute value of the ratio of the estimated to the known (i.e. Column 2/Column 1) which is transformed with a logarithm (base 2). Successive columns (5, 7, 9, 11, 13, 15, 17) use the preceding estimation value.

Table 2 lists all 18 of the scaling populations used in this study. For each, the known population size is listed in column 1 and the back estimated population size using the original estimator is listed in column 2. A visual comparison of the numbers quickly reveals that some of the estimates are sizably different. For example, the NSUM back estimate for firefighters in Nebraska shows a value of over 18,000, while data from the Bureau of Labor Statistics (which was used in Eq 2 to calculate personal network size) suggests that there are only 1,200. Other back estimates are less drastically divergent, such as the number of people named Adam in Nebraska (which is estimated to be 4,754 while information from the Census suggests that there are 4,839).

Following Guo and colleagues [12], we can compare the performance of the estimators by using the ratio of the back estimates to the known values for each subpopulation. A larger (or smaller) ratio indicates a greater difference between the back estimate and the known population. Such a measure provides a scale from 0 to infinity, with an ideal value of 1 (where the back estimate, based on all of the scaling variables together, is equal to the known value). In Guo and colleagues [12] study, a correction process was employed that discarded any scaling variable from the estimation process if the ratio of estimated to known exceeded 2 or was below 0.5. Using the over/under of 0.5 and 2, these authors eliminated 11 of their 19 total predictors at once, using the remaining 8 as the basis for their actual estimates of their target population.

Unfortunately, a one-step trimming process misses the fact that the elimination of any single scaling variable will necessarily change the estimate of personal network size, and thus the back estimation of all of the other scaling variables. Under such conditions, viewing the performance of the scaling variables as fixed regardless of their combination seems problematic. In place of this one step elimination process, we propose a recursive process of repeatedly removing the worst performing scaling variable, in light of the results of all those remaining at any given stage of the process. Rather than a flat cutoff point, we instead use the log base 2 of the ratio of back-estimated to known (in essence, the log of Guo et al’s ratio [12]). This transformation produces a performance metric that is continuous and symmetrical around zero. The absolute value of this number indicates that the greater the value, the greater the distance between the back estimate and the known population of the particular scaling variable regardless of which is larger.

Looking down column 3 of Table 2 it is apparent that the greatest difference between back estimates and known populations for the 18 scaling variables is the number of firefighters in Nebraska, with a value of 3.93. Discarding firefighters from our estimation equation is done by returning to Eq 2, which calculates the respondent’s personal network size, and removing the firefighters count and known population from the estimation process. We then recalculate the personal network size and rerun the back estimates for the scaling variables using Eq 3. The new back-estimates are shown in column 4 of Table 2, labeled as IT1 (Iteration 1). By repeating the process of calculating the ratio of estimates to known populations, applying base 2 logarithms, and then comparing the absolute values of the result, we can find the next most extreme variable from the list of knowns. In this case the difference in the NSUM estimate for the number of U.S. Postal Carriers in Nebraska and the known population is the greatest (with a value of 1.77). Removing the Postal Carrier estimator from Eq 2 and recalculating personal network size and new back-estimates sets us up for the next round of calculating distance.

This process is carried out recursively, until the absolute value of the log distance between estimates and known values is below one for all remaining predictors. The choice of log base 2 absolute value of 1 means that in the ratio of back estimated population to known value, the denominator is no more than twice the size of the numerator, and vice versa—or in other words, that the ratios are the same cutoff used by Guo and colleagues [12] (i.e. 0.5 and 2). Other cut thresholds might be chosen. The point here is that the recursive process allows for the reconsideration of the performance of every scaling variable in light of all those remaining. Such a process allows for the fact that scaling variables may not be independent, but rather may contain complex interdependencies. According to the initial back estimation at step 0, firefighters, Walter, Martha, and correctional officers performed the most poorly. Yet once the firefighter variable is removed, postal carriers become the most problematic. In the third round airline pilots have the greatest distance between known and estimated values. Finally, at round four, Martha is eliminated. The second and third eliminations (postal carriers and airline pilots) were not among the top 4 worst predictors. Had we used the one step, block elimination process, a different set of variables would have been eliminated. This (radical) reordering at each step illustrates how the distance measure changes across iterations and why the bulk removal process could result in removing variables that would not actually need to be eliminated from the calculation. This is particularly important for researchers who have a limited number of predictors and are trying to make difficult decisions about which variables to cut for the best results.

The recursive trimming process also changes the target population estimates at each step. Using the original estimator, we went through seven iterations until the distance metric for all remaining predictors was below a value of one, as shown by Table 2. Table 3 then shows the differences in the population estimates before the recursive process was begun, and the estimates after completing seven iterations. All three of the estimates increased sizably and by the same factor (1.26). The recursive process will thus have a larger raw effect on population estimates of larger numeric size. We note as well that the estimate of average personal network size decreased from 604.03 to 464.28 by the end of the recursive process, accompanied by a decrease in the variance and maximum size of a respondent's personal network as well.

**Table 3:** Change in Three Population Estimates and Personal Network Size over the Recursive Trimming Process through Seven Iterations using the Original Estimator.

		IT0		IT7	
		Estimate (s.e)	95% CI	Estimate (s.e)	95% CI
Moved to Nebraska from within the US in last 2 years		12184	[11673, 12695]	15407.53	[14761, 16053]
		(260.60)		(329.54)	
Would not approve of interracial dating		17892	[17273, 18510]	22624.97	[21842, 23408]
		(315.79)		(399.33)	
Heroin use in last 30 days		368	[279, 457]	465.19	[353, 577]
		(45.28)		(57.26)	
Personal Network Size					
	Mean	604.03		464.28	
	Standard Deviation	694.04		444.82	
	Min	0.00		0.00	
	Max	5944.31		4185.61	
	N	555.00		571.00	

## Incorporating Weights into the Network Scale-up Estimator

One of the strengths of the network scale-up estimator is that it can take advantage of sampling frames with known sampling probabilities to estimate the size of hidden and hard-to-reach populations. These sampling frames also allow for the incorporation of sampling weights into the population estimation process. Weights can adjust for probability of selection, survey nonresponse, and allow for post-stratification adjustments in order to make the sample more representative to the population. Using weights would theoretically result in better target population estimates and the possibility of using NSUM techniques successfully with more complex sampling designs.

Weights can be added to both the original and the MoS estimation formulas with little trouble, modifying Eqs 3 and 9 respectively. In both cases the number of people known by the respondent in a given subpopulation (*m*_*ij*_) is multiplied by the individual’s final weight (*w*_*i*_). Eq 10 shows the weighted formula for the original estimator and Eq 11 shows the weighted formula for the MoS estimator. For the current data we are using a weight which adjusts for the probability of selection within a household and then post-stratifies the sample to match the distribution of sex and age for Nebraska. The selection weight is necessary as we used address based sampling which randomly selects households in Nebraska which may then have multiple potential survey respondents. Post-stratification by sex and age helps adjust our estimates to match population distributions.

$${\hat{e}}_{j}=\frac{\sum_{i}m_{ij}w_{i}}{\sum_{i}{\hat{c}}_{i}}t$$ (10)

$${\hat{e}}_{j}=\frac{\sum_{i}\frac{m_{ij}w_{i}}{{\hat{c}}_{i}}}{i}t$$ (11)

Table 4 shows how the three population estimates and the estimated personal network size change when weights are incorporated in both the original and MoS estimators. Sizable changes are seen in all three of the population estimates. According to both estimators, the number of people who moved to Nebraska from another state in the U.S. increases considerably. Compared to the 2 year ACS total of 89,120, the MoS estimator still performs better (providing a weighted estimate of 114,929 people, compared to the weighted original estimate of 16,232). Although the weighted MoS estimator is now overestimating the population size compared to the ACS numbers, it is still considerably closer than the estimate provided by the weighted original estimate for the same population. The number of Nebraskans who do not approve of interracial dating also increased compared to the unweighted estimates using the original estimator, but decreased according to the MoS. In both cases, the number of people who used heroin in the last 30 days decreased.

**Table 4:** Change in Three Population Estimates and Personal Network Size without Recursive Trimming over the Original and MoS Estimator, with Weights and Without.

		Original				MoS			
		Unweighted		Weighted		Unweighted		Weighted	
		Estimate (s.e)	95% CI	Estimate (s.e)	95% CI	Estimate (s.e)	95% CI	Estimate (s.e)	95% CI
Moved to Nebraska from within the US in last 2 years		12184	[11673, 12695]	16232	[15643, 16822]	75800	[74821, 76777]	114929	[113724, 116133]
		(260.60)		(300.79)		(499.14)		(614.62)	
Would not approve of interracial dating		17892	[17273, 18510]	19234	[18592, 19876]	22614	[22079, 23148]	19655	[19157, 20153]
		(315.79)		(327.42)		(272.63)		(254.17)	
Heroin use in last 30 days		368	[279, 457]	288	[210, 367]	454	[379, 530]	346	[280, 412]
		(45.28)		(40.09)		(38.64)		(33.73)	
Personal Network Size									
	Mean	604.03		604.03		1024.28		1024.28	
	Standard Deviation	694.04		694.04		1559.17		1559.17	
	Min	0.00		0.00		0.00		0.00	
	Max	5944.31		5944.31		16794.19		16794.19	
	N	555.00		555.00		555.00		555.00	

## Testing All Three Components

In the final stage of this article we combine the above elements (MoS estimator, recursive back estimation trimming, and the use of population weights) along with the removal of outliers to produce final population estimates, in what we feel is a significant step forward in the NSUM estimation procedure. Table 5 shows the final size estimates for our three subpopulations in Nebraska. Moving from left to right we first display the unweighted and then weighted estimates using the original estimator, and then the unweighted and weighted estimates using the MoS estimator. All four estimates have gone through the recursive trimming process proposed here to remove poor scaling variables. Each estimate has also gone through an outlier removal process wherein we remove estimates of personal network size that are greater than 1.5 times the interquartile range plus the value of the 75^th^ percentile, or less than 1.5 times the interquartile minus the value of the 25^th^ percentile.

**Table 5:** Changes in Final Population Estimates and Personal Network Size between Original/MoS and Unweighted/Weighted Proceedures after Recursive Trimming.

		Original				MoS			
		Unweighted		Weighted		Unweighted		Weighted	
		Estimate (s.e)	95% CI	Estimate (s.e)	95% CI	Estimate (s.e)	95% CI	Estimate (s.e)	95% CI
Moved to Nebraska from within the US in last 2 years		16039	[15310, 16768]	21390	[20578, 22202]	64320	[63067, 65573]	90073	[88631, 91515]
		(371.99)		(416.58)		(639.30)		(735.76)	
Would not approve of interracial dating		22734	[21867, 23602]	23883	[23025, 24742]	21907	[21175, 22638]	21250	[20550, 21951]
		(442.87)		(440.19)		(373.10)		(357.37)	
Heroin use in last 30 days		535	[402, 668]	404	[433, 516]	385	[288, 481]	281	[200, 361]
		(67.93)		(57.28)		(49.43)		(41.08)	
Personal Network Size									
	Mean	397.38		423.37		556.93		584.39	
	Standard Deviation	278.23		293.65		453.98		486.36	
	Min	0.00		0.00		0.00		0.00	
	Max	1243.25		1313.92		1988.66		2100.17	
	N	545.00		544.00		528.00		532.00	

The estimates for the number of people who have moved to Nebraska from another state in the U.S. vary considerably across all four procedures: 16,039; 21,390; 64,320; and 90,073 respectively. Summing the ACS totals for the same type of migration during 2012 and 2013 gives us a total of 89,120. Each improvement we made to the NSUM process brings us closer to the ACS figure, but the combination of all three elements discussed in this paper provides a population estimate of 90,073 with 95% confidence intervals that include the ACS estimate of 89,120. Estimates for disapproval of interracial dating (22,734; 23,883; 21,907; 21,250) and heroin use in the last 30 days (535, 404, 385, 281) vary considerably across estimation methods as well. The accuracy of the estimate of state-to-state migration verified by the ACS data suggests that the final estimates for those who disapprove of interracial dating and who have used heroin in the last 30 days are more accurate when using all three of our proposed methods as well.

The MoS estimator, used in conjunction with the recursive trimming process, also preserves the largest number of scaling variables. The original NSUM estimator requires the discarding of seven scaling variables before meeting the distance threshold, even given the recursive trimming process. The MoS estimator discards only four. Keeping as many scaling variables as possible in the estimation process is highly recommended for robust estimations. This becomes far more important when a researcher is limited in space and resources and can only field a small number of scaling variables.

Personal network size varies considerably between different estimation forms in Table 5. Average network size ranges from 397.38 to 584.39 a range of maximum size between 1243.25 and 2100.17. Compared to the initial estimates of personal network size shown in Table 4 there has been a considerable decrease in both average and maximum network size. Although the recursive process does reduce the estimate of personal network size, the majority of the change between Table 4 and Table 5 is attributed to removing outliers after the recursive trimming process is completed.

## Conclusion

The network scale-up method is an important tool in the study of hidden and hard-to-reach populations. Its ability to generate accurate estimates of these populations using conventional sampling frames and survey techniques allows for data collection efforts that are considerably cheaper and faster than commonly used techniques to study hidden populations. Developing new improvements to the NSUM estimation process is important as the method begins to become more popular in new areas of the world and is applied to new populations.

We propose three adjustments to the original implementation of the network scale-up method. First, changing the estimation equations to take into account the mean of sums of ratios instead of the ratio of the sums preserves a respondent’s exposure to each scaling subpopulation and allows these differences to exert equal weight upon the estimates. These changes are simple conceptually, but as shown, can have considerable effect upon population estimates generated by the NSUM estimator (see Table 5).

Second, we introduce means to incorporate sampling and post-stratification weights into the NSUM estimation process. Building weights into the equations allows researchers to take advantage of the sampling frames and their respective weighting adjustments which are seldom available to those interested in hidden and hard-to-reach populations. As shown above, neglecting to include weights in NSUM estimation ignores an important source of data correction that can greatly improve NSUM population estimates.

Third, we discuss the benefits of using back estimation in a recursive fashion to improve population estimates. Instead of removing poor predictors in bulk, we suggest removing the most egregious predictor and then rerunning the back estimates. This process recognizes the dependency of the back estimates upon all the predictors that are used in the method. Removing poor estimators singly and in a recursive fashion allows researchers to examine how the removal of each estimator affects the other results.

We note that this recursive process provides an important check on scaling variables. A difference in performance across individual variables does not necessarily indicate problems with the estimation technique. Rather, it more likely reveals a measurement error or poor question design. If we consider the example of the firefighters in our own survey, we find a wide discrepancy between the “known” value of 1,200 firefighters in Nebraska and the original back estimate of 18,000. We speculate that this discrepancy likely represents a poorly phrased question. The number of firefighters which we obtained from the Bureau of Labor Statistics (1200) represents professional and paid firefighters. Our survey did not specify that the firefighters needed to be paid professionals in order to match the criteria used by the BLS (and in retrospect, this may not have made that much of a difference). This is important because Nebraska is predominantly a rural state and thus has a sizeable portion of volunteer firefighters. These volunteers would not be represented in the BLS statistic, but would likely be identified as firefighters by our respondents. Because we did not correctly phrase our question, respondents were free to include anyone whom they considered a firefighter, professional or volunteer, significantly inflating the number of “knowable” firefighters in the population. This provides an unfortunately apt example of why pre-testing surveys and conducting cognitive interviews can eliminate considerable problems after a survey is complete [23,24]. The fact that this error can be discovered (and corrected) by the back estimation procedure described here provides something of a safety net for situations where large scale pre-testing is not possible.

Establishing how many predictors to cut, and where an appropriate threshold point for stopping the recursive trimming process may be, is likely to be highly dependent upon the characteristics of the NSUM project. Studies using larger numbers of scaling variables can afford to trim all those that are suspect. However, when there are fewer total predictors, say less than 5–8, over trimming of scaling variables can potentially mask variation across the respondent pool. Researchers will need to balance the desire to remove inaccurate predictors with the need to maintain sufficient variation in the variables that are used in the estimation of personal network size. In these situations the recursive method becomes more important as analysts seek to eliminate the most egregious predictors while maintaining as many scaling variables as possible.

Our estimate of the number of people who have moved into Nebraska in the last 2 years from another U.S. state indicates that the estimation procedure changes introduced here provide a significant improvement over a one-step estimation procedure, and over the original estimator. This also gives us greater confidence in our other target population estimates, which are not as easily checked through verifiable sources. The implications of this new estimation procedure for previously estimated target populations may be a worthwhile question for researchers that have already carried out their own NSUM data collection. Looking ahead, the recursive back estimate trimming process may encourage researchers to rethink how many scaling variables they choose, and widen the potential list of these variables now that questions of equivalent size are less significant. Together this adds greater flexibility to the NSUM method, even as early results indicate that it also improves accuracy.
